# Uncertain deduction and conditional reasoning

**DOI:** 10.3389/fpsyg.2015.00398

**Published:** 2015-04-08

**Authors:** Jonathan St. B. T. Evans, Valerie A. Thompson, David E. Over

**Affiliations:** ^1^School of Psychology, University of PlymouthPlymouth, UK; ^2^Department of Psychology, University of SaskatchewanSaskatoon, SK, Canada; ^3^Department of Psychology, Durham UniversityDurham, UK

**Keywords:** uncertain premises, conditional reasoning, new paradigm psychology of reasoning, p-validity, coherence, explicit inference, fallacy

## Abstract

There has been a paradigm shift in the psychology of deductive reasoning. Many researchers no longer think it is appropriate to ask people to assume premises and decide what necessarily follows, with the results evaluated by binary extensional logic. Most every day and scientific inference is made from more or less confidently held beliefs and not assumptions, and the relevant normative standard is Bayesian probability theory. We argue that the study of “uncertain deduction” should directly ask people to assign probabilities to both premises and conclusions, and report an experiment using this method. We assess this reasoning by two Bayesian metrics: probabilistic validity and coherence according to probability theory. On both measures, participants perform above chance in conditional reasoning, but they do much better when statements are grouped as inferences, rather than evaluated in separate tasks.

## Introduction

### Paradigm shift in the psychology of reasoning

The psychology of deductive reasoning is undergoing a paradigm shift, which is the consequence of the introduction of Bayesian approaches into the field (see Oaksford and Chater, 2007, 2010; Over, 2009; Manktelow et al., 2011; Elqayam and Over, 2012; Evans, 2012; Baratgin et al., 2013, 2014). In the real world, there are few propositions that people can hold are certainly true, or certainly false, and most of their beliefs come in degrees, which are technically subjective probabilities. We may believe that a grant application has a 50-50 chance of success, or that we will probably be happier if we take a promotion with more responsibility, or that we are unlikely to get on with the new boss we met this morning. It is precisely such uncertain beliefs that we need to take into account when making decisions and solving problems in everyday life. Essential to this process is the ability to combine uncertain beliefs and draw inferences from them, and this is what the new psychology of reasoning is concerned with studying.

The method of study that dominated the field for 40 years or so is the traditional *binary deduction paradigm* (Evans, 2002), inspired by extensional logic and intended to test whether people were capable of logical reasoning without formal training. With this method, participants are given the premises of a logical argument, instructed to *assume that they are true*, and asked to decide whether a purported conclusion *necessarily* follows. They were expected to answer “yes” for arguments considered valid in extensional logic and “no” for those considered invalid. Thus, measured, however, logical reasoning is observed to be generally poor and subject to various cognitive biases (for recent reviews, see Evans, 2007; Manktelow, 2012).

We believe that this traditional paradigm maps quite poorly on to the requirements of real world reasoning. Two key features of the method, which directly reflect the classical binary logic used to assess the accuracy of reasoning, are the instruction to assume the premises and the classification of all statements as simply true or false. High expertise in assumption-based reasoning generally requires specialized training and when logical problems of this kind are administered to naïve participants, we find it unsurprising that error rates are high. We also note that such reasoning loads heavily on working memory and that those of high intelligence do better at these tasks (Evans, 2007; Stanovich, 2011). But everyday reasoning cannot be a specialized tricky business requiring elite professionals and condemning the majority to mistakes. If that were the case, then most people would be incapable of intelligent actions. For these reasons, a number of authors have questioned the relevance of extensional logic and the standard deduction paradigm based upon it (e.g., Oaksford and Chater, 1998, 2007; Evans, 2002; Evans and Over, 2004; Pfeifer and Kleiter, 2010). The new approach treats reasoning as concerning degrees of belief, rather than assumed truth and falsity, and allows that inferences can be drawn with a varying degrees of confidence (Oaksford and Chater, 2007; Evans and Over, 2013).

What is yet to emerge, however, is a clear alternative method for studying reasoning to the traditional deduction paradigm. There are a number of studies which have relaxed instructions, so that participants are given premises but not instructed to assume that they are true, and in which they are sometimes permitted to express degrees of confidence in the conclusions. These are generally known as pragmatic reasoning instructions. Such instructions have been applied to one of the most commonly studied tasks, that of conditional inference. Participants are presented with a conditional and asked whether conclusions follow for four simple inferences, two of which are considered, in most normative systems, as logically valid and two invalid. See Table 1.

**Table 1:** **The four conditional inferences commonly studied by psychologists**.

Modus ponens	MP	If p then q; p therefore q	Valid
Denial of the antecedent	DA	If p then q; not-p therefore not-q	Invalid
Affirmation of the consequent	AC	If p then q; q therefore p	Invalid
Modus tollens	MT	If p then q; not-q therefore not-p	Valid

When the traditional binary paradigm and abstract materials are used (e.g., If the letter is A then the number is 5), participants only show good logical performance on MP, which is nearly always endorsed. MT is also valid but is not endorsed as often as MP, and AC and DA are commonly endorsed, despite being invalid (Evans and Over, 2004). When realistic content is introduced, however, this can substantially affect responding. It has been known for some years that people may resist the simple valid inference MP when they disbelieve the conditional statement (George, 1995; Stevenson and Over, 1995; Politzer, 2005). For example, given the argument

If the UK builds more nuclear power plants the environment will be safer. (1)
The UK will build more nuclear power plants.
Therefore, the environment will be safer.

Many participants will say that the conclusion does not follow, despite the obvious logic. As the early studies also showed, the exact nature of the instructions is critical. If strict traditional reasoning instructions are employed, with participants asked to assume the premises, they are more likely to resist belief influences and reinstate the inference. However, a recent study has shown the ability to suppress the influence of prior belief on conditional reasoning is restricted to those of higher cognitive ability, even within a university student population (Evans et al., 2010). This difference only occurred under traditional deductive reasoning instructions; with pragmatic reasoning instructions, high ability participants were equally belief “biased.” These findings (and many others) suggest to us that assumption-based reasoning is a form of effortful hypothetical thinking (Evans, 2007; Evans and Stanovich, 2013). Belief-based reasoning by contrast is an everyday, natural mode of thought that requires little effort.

If participants are to be allowed to express uncertainty in their conclusions, then are we still studying deduction, or is this a form of inductive inference? In a recent paper, we have shown that deduction in the new paradigm is still distinct from inductive reasoning, but it is described better as what we call *uncertain deduction* (Evans and Over, 2013; see also Pfeifer and Kleiter, 2011). That is, people make deductions in which the uncertainty of the premises is reflected (rightly, according to probability theory) in the uncertainty of the conclusion. Consider a famous piece of reasoning by Sherlock Holmes (see Table 2).

**Table 2:** **Extract from Conan-Doyle's, *The Sign of Four* (1890)**.

(HOLMES TO WATSON) “Observation shows me that you have been to the Wigmore Street Post-Office this morning, but deduction lets me know that when there you dispatched a telegram.”
“Right!” said I. “Right on both points! But I confess that I don't see how you arrived at it. It was a sudden impulse upon my part, and I have mentioned it to no one.”
“It is simplicity itself,” he remarked, chuckling at my surprise,–“so absurdly simple that an explanation is superfluous; and yet it may serve to define the limits of observation and of deduction. Observation tells me that you have a little reddish mold adhering to your instep. Just opposite the Wigmore Street Office they have taken up the pavement and thrown up some earth which lies in such a way that it is difficult to avoid treading in it in entering. The earth is of this peculiar reddish tint which is found, as far as I know, nowhere else in the neighborhood. So much is observation. The rest is deduction.”
“How, then, did you deduce the telegram?”
“Why, of course I knew that you had not written a letter, since I sat opposite to you all morning. I see also in your open desk there that you have a sheet of stamps and a thick bundle of post-cards. What could you go into the post-office for, then, but to send a wire? Eliminate all other factors, and the one which remains must be the truth.”

Conan Doyle always used the term “deduction,” but many readers may have wondered whether the reasoning described is not some type of non-demonstrative inference, such as an abductive inference to the best explanation of the evidence. The conclusions always seem to have a degree of uncertainty (despite being rarely mistaken in the stories). We do not deny that some of Holmes' reasoning is inductive or abductive, and Conan Doyle himself may not have had a very precise understanding of what “deduction” means. But focus on the final sentence above: “Eliminate all other factors, and the one which remains must be the truth.” The form of reasoning referred to here is the *disjunctive syllogism*: the logical inference to *q* from the premises *p or q* and *not-p*. Two “factors,” *p* and *q*, are referred to in *p or q*, and *not-p* “eliminates” one of these, leaving *q* as what “must” follow. In the story, *p or q* is Watson going to Wigmore Street to send a letter or a wire, and *not-p* is not going there to send a letter, with sending a wire as the conclusion. This inference is clearly deductive, but of course both *p or q* and *not-p* are uncertain to a degree, and the conclusion falls short of certainty. Wigmore Street is just around the corner from Baker Street, and Watson could have gone out for any number of reasons that would have placed him “opposite” the post-office there.

In this example, Holmes' disjunctive syllogism is *classically valid*, in that its conclusion must be true given that its premises are true, but it is not necessarily *sound*. A sound inference is a valid inference the premises of which are actually true. In other words, we can only be sure of the conclusion if we are sure of the premises. The problem with the classical notion of soundness is that, like classical validity, it is black and white. An argument is either sound or it is not. We might feel some doubt that Holmes' argument is sound, but we are losing something if we totally disregard it. His premises are plausible, and his conclusion is more likely than not. In an uncertain world, that is better than nothing. The new paradigm is really an extension of the old that can deal not just with contexts where statements can be assigned probabilities of 1 (“true”) or 0 (“false”), but all values in between. We cannot usually be certain of our premises and conclusions, and have to ask what other degrees of confidence we should have in them. Classical logic does not provide a means for doing this, and we must look elsewhere. The obvious place is in Bayesian subjective probability theory, which extends classical logic in precisely this manner.

### Normative assessment of uncertain deduction

The binary and extensional logic of the old deduction paradigm has no means of evaluating inferences from uncertain premises. However, two Bayesian standards, which we have discussed previously (Evans and Over, 2013), can be applied. The first is probabilistic validity, or *p-validity* (Adams, 1998; see also Gilio, 2002; Gilio and Over, 2012). Probabilistic validity is a generalization of classical validity. The latter is truth-preserving. The conclusion of a classically valid inference will be true given that the premises are true: one cannot go from truth in the premises to falsity in the conclusion. Similarly, p-valid inferences are probability-preserving. One cannot go from high probability in the premise of a p-valid single premise inference to low probability in the conclusion. For example, the inference of *and-elimination*, inferring *p* from *p and q*, is p-valid because *P(p and q)* ≤ *P(p)* for all coherent probability assignments. People commit the conjunction fallacy when they violate the p-validity of this inference (Tversky and Kahneman, 1983).

The matter is a bit more complex for inferences with two or more premises. There is a problem of specifying how the probabilities of two or more premises are to be combined, but this is avoided by saying that a p-valid inference cannot take us from low uncertainty in the premises to high uncertainty in the conclusion. We define the *uncertainty* of a proposition *p* as one minus its probability, 1—*P(p)*. Then an inference with two or more premises is p-valid if and only if the uncertainty of its conclusion is not greater than the sum of the uncertainties of its premises for all coherent probability assignments. A p-valid deduction from premises cannot increase the uncertainty in the premises; it differs from induction in precisely this respect (Evans and Over, 2013)^1^. In Table 1, MP and MT are p-valid inferences, and AC and DA are p-invalid inferences.

To illustrate with conditionals, consider two sets of assignments of probabilities to the premises of an instance of the p-valid inference MP, inferring *q* from the premises *if p then q* and *p*:

**Table:** 

	A	B
*if p then q*	0.8	0.2
*p*	0.9	0.1

Consider set A first. The sum of the uncertainties of the premises of A is (1 − 0.8) + (1 − 0.9) = 0.3. The uncertainty of the conclusion should not exceed that limit, which implies that we would violate p-validity if we assigned a probability to the conclusion *q* of less than 0.7. In that case, we would be in violation of this Bayesian norm by being more uncertain of the conclusion of a p-valid inference than we were of the premises. The formal definition of the p-validity interval for the conclusion probability is shown in Table 3. As the uncertainty of the premises increases, the minimum probability value that can be assigned to the conclusion drops. Turning to B, we see that the uncertainties, 0.8 and 0.9, sum to 1.7. Whenever this figure is one or more, it means that we may assign *any* probability between 0 and 1 to the conclusion without violating p-validity. In other words, where premises have low degrees of belief, p-validity can never be violated. This is clearly something that researchers need to take into account. But there is a parallel with the classical position. When we judge that the premises of MP are false, we cannot violate classical validity by holding that the conclusion is also false, because we are not claiming that the conclusion is false when the premises are true.

**Table 3:** **Permitted intervals for conclusions probabilities for the four conditional inferences on two measures**.

**p-validity**			**Coherence**	
**Inference**	**Min**	**Max**	**Min**	**Max**
MP	max{x+y−1,0}	1	xy	1−y+xy
DA	max{x+y−1,0}	1	(1−x)(1−y)	1−x(1−y)
AC	max{x+y−1,0}	1	0	min{y/x,(1−y)/(1−x)}
MT	max{x+y−1,0}	1	max{(1−x−y)/(1−x),(x+y−1)/x}	1

Notes: (1) In each case x = The probability of the major premise, if p then q, and y = the probability of the relevant minor premise, i.e., P(p) for MP, P(not-p) for DA, P(q) for AC, and P(not-q) for MT.

(2) P(if p then q) = P(q|p) is assumed for calculation of the coherence but not p-validity intervals.

(3) For both measures, a “hit” is defined as an estimated conclusion probability which is between the minimum and maximum values shown in the table.

A further important point about p-validity to stress is that it is defined in terms of coherent probability assignments. For conditional inferences, this coherence depends on the probability of the natural language conditional, *P(if p then q)*. There has been much debate in logic, philosophy, and psychology about this probability (Edgington, 1995; Evans and Over, 2004). One possibility is *P(if p then q)* is the probability of the material conditional of elementary extensional logic, *P(not-p or q)*. If this is so, then the assignments *P(if p then q)* = *P(not-p or q)* = 0.8, *P(p)* = 0.9, and *P(q)* = 0.7 are coherent. Another possibility is that *P(if p then q)* is the conditional probability of *q* given *p*, *P(q|p)*, and if this is so, *P(if p then q)* = *P(q|p)* = 0.8, *P(p)* = 0.9, and *P(q)* = 0.7 are incoherent. In fact, making the latter probability judgments is equivalent to the conjunction fallacy, since *P(p and q)* = *P(p)(q|p)* = 0.72 and yet *P(q)* is judged to be 0.7. There are still other possibilities for conditionals based on possible-worlds semantics (Evans and Over, 2004). Nevertheless, judging *P(if p then q)* = 0.8, *P(p)* = 0.9, and *P(q)* < 0.7 is incoherent for all these possible conditionals and violates p-validity, by increasing uncertainty in the conclusion of an inference, MP, which is p-valid for both interpretations of the conditional. To make our study of p-validity as general as possible, and to presuppose as little as possible, we do not make any special assumption about *P(if p then q)* in our study of p-validity. We will simply ask whether people conform to p-validity by making the uncertainty of the conclusion in a conditional inference less than or equal to the sum of the uncertainties of the premises, and whether they conform more to p-validity when they are given explicit inferences. We ask these questions about both the normatively p-valid inferences of MP and MT, and the normatively p-invalid inferences of AC and DA. As we have noted above, people often endorsed AC and DA as “valid” inferences in traditional studies in the binary paradigm, and we wished to test whether they would also do in a probabilistic study.

There are certainly strong arguments (Edgington, 1995) that the probability of the natural language indicative conditional is the conditional probability, that it satisfies what has been called *the Equation*, *P(if p then q)* = *P(q|p)*. If the Equation holds, the appropriate normative rules for degrees of belief about the natural language conditional are those for conditional probability in Bayesian probability theory. There is much empirical evidence to support the Equation as descriptive of most people's probability judgments (Douven and Verbrugge, 2010; e.g., Evans et al., 2003; Oberauer and Wilhelm, 2003; Over et al., 2007; Politzer et al., 2010; Fugard et al., 2011; Singmann et al., 2014). The majority of participants respect the Equation, but this is by no means universal. It is also found more often in those of high cognitive ability (Evans et al., 2007). The evidence supporting the Equation is at its strongest for the type of realistic conditionals used in our experiment below (see Supplementary Material and Over et al., 2007; Singmann et al., 2014), but we will still not assume that *P(if p then q)* = *P(q|p)* in our study of p-validity, for the reason already given.

The second Bayesian standard we will use to assess deduction from uncertain premises is coherence itself. Here our method does presuppose the Equation, *P(if p then q)* = *P(q|p)*, for otherwise we cannot lay down precise conditions for the coherence of inferences that contain conditionals. We could use “p-consistent” for this generalization of binary consistency (and have done so in Evans and Over, 2013), but p-consistency has been defined in more than one way (Adams, 1998, p. 181), and “coherence” is standard in judgment and decision making. Degrees of belief and subjective probability judgments are coherent when consistent with the axioms of probability theory. Degrees of beliefs in different statements that relate to each other in some way may or may not be coherent. As we saw above, people are incoherent and make judgments equivalent to the conjunction fallacy if they judge that *P(p and q) > P(p)*. In commenting upon this fallacy, Tversky and Kahneman (1983, p. 313) stated that “… the normative theory of judgment under uncertainty has treated the coherence of belief as the touchstone of human rationality.” Their findings have stimulated a rich literature on this fallacy and its possible explanation in terms of the representativeness heuristic (see Tentori et al., 2013, for a recent contribution). Our question in this paper is not whether people are coherent in their conjunction inferences, but rather whether they are coherent in their conditional inferences, and whether their coherence is increased when the conditional inferences are made explicit.

In our approach, *P(q|p)* is not necessarily given by the ratio, *P(p and q)/P(p)*, but rather by the *Ramsey test* (Edgington, 1995; Evans and Over, 2004). Using this “test” on *if p then q*, we hypothetically suppose that *p* holds, while making suppositional changes in our beliefs to preserve consistency, and then make a judgment about *q*. This procedure allows us to infer a value for *P(q|p)* when *P(p)* cannot be fixed because *p* refers to an action which we are trying to make a decision about, and even when we judge that *P(p)* = 0 (see also Gilio, 2002; Pfeifer and Kleiter, 2009, 2010, 2011; Gilio and Over, 2012).

To illustrate our approach, with the Equation now assumed, suppose we want to make a probability judgment about the conditional, “If Dr Adler submits her paper to the Journal of Psychology Reports, it will be accepted.” We would use the Ramsey test and suppose that she does make the submission, and then using our knowledge of her ability and the standards of the journal, we would make a judgment about the probable acceptance of her paper. Suppose the result is a degree of belief of 0.8 that it will be accepted under that supposition, and with *P(if p then q)* = *P(q|p)*, our degree of confidence in the conditional will be 0.8. When we take it as certain that Dr Adler will submit her paper to the journal, we should believe 0.80 that it will be accepted, and any other figure would be incoherent. If, however, we have some uncertainty about whether she will submit there or to a journal we have no knowledge of, it becomes more complicated. Suppose we believe only 0.50 that she will submit to the Journal of Psychological Reports and will otherwise submit to the unknown journal. Now we cannot give a specific probability to the paper being accepted, for we lack information about the unknown journal and it acceptance rate.

It is important to understand that in a case like this our belief in the statement “Dr Adler's paper will be accepted” is still constrained. It has to fall within a range of probability values in order to be coherent. Consider the two extreme cases. At one extreme, if Dr Adler submits to the unknown journal, it is certain that the paper will be accepted. So there is a 0.50 × 0.80 plus a 0.50 × 1 chance of the paper being accepted, which is 0.90. At the other extreme, it is certain that the paper will be rejected at the unknown journal: now the chance is just 0.50 × 0.80 = 0.40 that the paper will be accepted. To be coherent, then, the probability we can set for the paper being accepted has to lie in the interval [0.4, 0.9]. Anything outside of this range is inconsistent with probability theory. Table 3 shows the formulae for computing this interval for both MP (the case considered) here, and the other three conditional inferences (see Pfeifer and Kleiter, 2009). Note that it is not just the valid inferences that are constrained by coherence. We can compute intervals for all four cases. A study has been reported testing participants for coherence with these equations (Pfeifer and Kleiter, 2010). We also do this but with a different experimental method, as described below.

A probabilistic theory of conditional inference has been presented by Oaksford and Chater (2007; see also Oaksford et al., 2000), and readers may wonder how this relates to the current analysis. These authors consider contexts in which the major premise of a conditional inference is uncertain, but the minor premise is certain. For example, Dr Adler might herself be certain where she will submit her paper. With *P(p)* = 1, the MP interval collapses to *P(q) = P(q|p)*, which is what Oaksford and Chater give as the probability of the conclusion of MP. Their equations for other inferences also take point values for the same reason. Note that participants who conform to Oaksford and Chater's equations will necessarily be in the intervals of Table 3. However, we cannot test conformity to their specific equations here, because the minor premises in our materials will rarely be certain (see also Oaksford and Chater, 2013, for an extension of their theory). Indeed, a key purpose of our study is to explore how people take into account the uncertainty in both premises when they reason with conditionals.

#### The study

In this study we examine the manner in which naïve participants will assign probabilities to both premises and conclusions of uncertain arguments. In view of the paucity of data on uncertain deduction our principal aim is to discover the extent to which such assignments conform to the two normative standards outlined above: probabilistic validity and coherence with probability theory. Pfeifer and Kleiter (2010) have already reported experiments on uncertain deductions, which they laid out as explicit conditional inferences (see also Singmann et al., 2014). They presented arbitrary premises with explicit probabilities attached and asked participants to indicate the range of probabilities within which the conclusion could fall. These could be compared with the normative equations shown in Table 3. They found generally good coherence for MP, but much poorer coherence for the other three inferences.

Our own method differs from of that Pfeifer and Kleiter in several ways. In place of premises with probabilities assigned by the experimenter, we used conditional statements concerning current affairs with evoke real world beliefs (see Supplementary Material). Probabilities were not assigned by the experimenter but taken from the participants themselves. We did this in two different ways. In a Belief group, participants assigned probabilities to the conditionals in one task—*P(if p then q)*—and to the relevant event probabilities in another—that is *P(p)*, *P(not-p)*, *P(q)*, and *P(not-q)*. This is not a reasoning task, of course, and thus can be used to measure what internal consistency, if any, is present in the beliefs expressed. This method has long been used in judgment and decision making, leading most famously to the discovery of the conjunction fallacy (Tversky and Kahneman, 1983) discussed above.

Our second method, more similar to that of Pfeifer and Kleiter (2010), was to lay out the statements as an explicit inference as in the following example:

GIVEN
If more people use sun screen then cases of skin cancer will be reduced
More people will use sun screen
THEREFORE
Cases of skin cancer will be reduced

Participants also assigned probabilities to the three statements here with the inferential structure now clearly cued. We differ from Pfeifer and Kleiter in that our participants provide their own premise probabilities and assign a point value, rather than an interval, to the conclusion. This method allows people to correct incoherence in their belief system as they can now reason explicitly about the way in which uncertainty in the premises should be reflected in the conclusion. We therefore expect stronger conformity to both p-validity and coherence measures in the Inference group.

### Method

#### Participants

Forty six undergraduate students of the University of Saskatchewan participated, with 23 assigned to each of the two experimental groups; Psychology students received course credits and others were paid for their participation.

#### Procedure

A set of 48 conditional statements were used concerning real world causal relations, similar to those in previous studies of the authors (Over et al., 2007) run on British participants, and known to vary widely in believability. All concerned causal relations in real world events, such as “If more people use sun screen then cases of skin cancer will be reduced.” Where necessary, the sentences were adapted to be relevant to the Canadian context. The sentences used are shown in Supplementary Material. The tasks were administered via computer software with the experimenter present. Participants were instructed that they would be receiving groups of statements which applied to Canada within the next 10 years, and that following each statement they would be asked to indicate the degree to which they believed the statement to be true (expressed as a percentage probability from 0 to 100%). All ratings were provided on a sliding scale located below each of the statements. Participants indicated their responses by clicking a bar on the scale and dragging it to the desired belief percentage. In the Belief group, participants assigned subjective probabilities to a randomized list of the 48 conditionals sentences and separately to a randomized list of the minor premises and conclusions corresponding to each sentence. For example, they gave probabilities for “more people will use sun screen,” “more people will not use sun screen,” “skin cancer rates will be reduced,” and “skin cancer rates will not be reduced” at some point in the list.

In the Inference group, as described above, participant's assigned probabilities to statements grouped as inferences with major premise, minor premise and conclusion rated in immediate succession with the whole argument visible. The headings GIVEN (before the premises) and THEREFORE (before the conclusion were also included). This resulted in another difference between the two groups: those in the inference group rated the same conditional sentence four times in different places as it appeared with each of the inference types, whereas in the belief group, each conditional sentence was rated only once. The order of presentation of each argument was fully randomized so that arguments using the same conditional statement could appear anywhere in the sequence.

### Results

As pointed out in the introduction, tests for p-validity are insensitive when the premise probabilities are low. For this reason, all analyses of p-validity reported are for a reduced set of 24 conditional sentences (mean = 60.6, *SD* = 11.3, Belief group ratings) with substantially higher degrees of belief in the major premise (conditional statement) than the other 24 (mean = 44.5, *SD* = 13.8). Coherence measures do not suffer from the same problem, so these analyses were conducted using the full set of 48 sentences.

#### Hit rates

Our first analyses concern the number of responses considered correct by our two main indices, p-validity and coherence. In each case we can define an interval within which the conclusion probability should be assigned. In case of p-validity, the conclusions have a maximum level of uncertainty, determined by the values actually assigned to the premises. This has to be computed separately for each participant and for each conditional sentence. In the example (A) discussed above, the maximum uncertainty of the conclusion was 0.3, meaning that the minimum probability value for the conclusion was 0.7. We call this value minP. A value of minP was computed from the premises for each participant problem and compared with the value actually assigned by the participant to the conclusion. Any value of minP or above was scored as making a “hit,” whether the inference was normatively p-valid, MP and MT, or not, AC and DA (as the participants might consider any of these inferences “valid”). Note that where the maximum uncertainty was 1 or more (as in example B above), minP was set equal to zero. (See Table 3 for formal definition of the correct interval for the conclusion probability.) A similar approach was used for the coherence analysis, except that here we need to compute two values for the conclusion—minP and maxP—using the equations shown in Table 3. Again this target interval depends on the actual probabilities assigned by each participant to each pair of premises. In the coherence analysis, any conclusion probability assigned in the interval [minP, max P] was scored as a hit. (Note that for any given problem minP is computed differently for p-validity than coherence and will not take the same value.)

**Figure 1:**
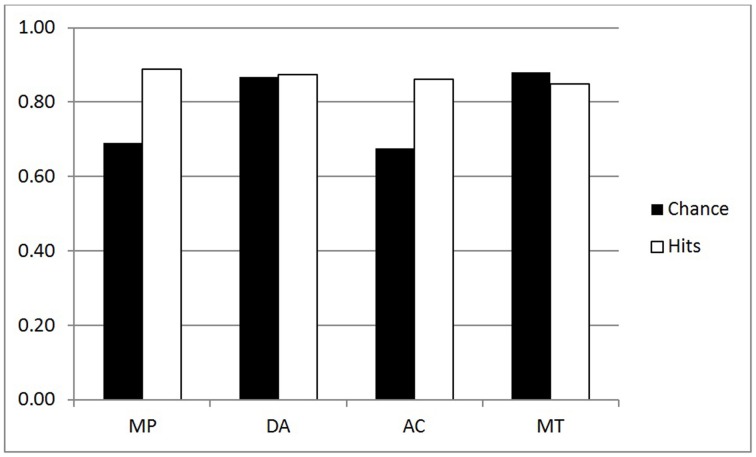
**p-validity analysis for the Inference group (Higher belief conditionals)**.

**Figure 2:**
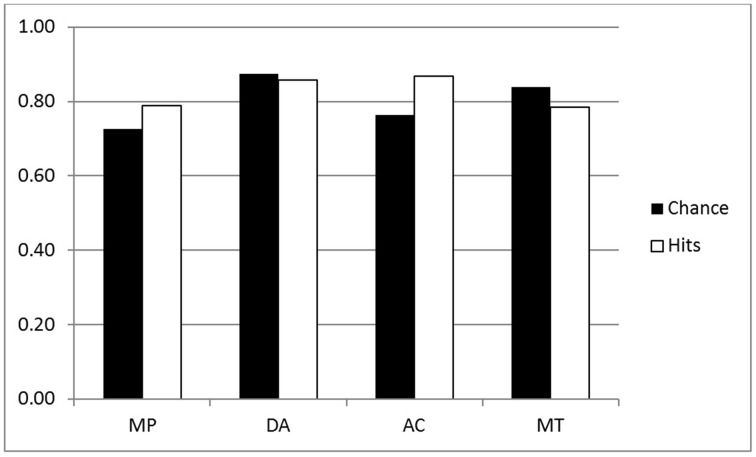
**P-validity analysis for the Belief group (Higher belief conditionals)**.

The frequency of hits for p-validity in the two groups are shown in the white bars of Figures 1, 2 (reduced set of higher belief conditionals); an analysis of the chance rates (black bars) is presented in a subsequent section. For the purpose of the ANOVA, we split the four inferences into two factors: Validity (MP, MT vs. DA, AC) and Polarity (MP, AC vs. DA, MT). The main purpose for doing this was to see more clearly whether classically defined valid inferences differed on our measures. In particular, we might expect greater conformity to p-validity on valid inferences, since p-validity is only normatively required for these. The ANOVA revealed several significant findings. As predicted, the Inference group had more hits (mean 0.87) than the Belief group (0.82) [*F*_(1, 44)_ = 4.27, MSE = 0.090, η^2^_*p*_ = 0.088, *p* < 0.05]. Contrary to expectations, however, invalid inferences (0.87) had significantly higher p-validity scores than valid inferences (0.83) [*F*_(1, 44)_ = 9.16, MSE = 0.064, η^2^_*p*_ = 0.172, *p* < 0.005]. There was also an interaction between the two factors [*F*_(1, 44)_ = 9.66, MSE = 0.067, η^2^_*p*_ = 0.180, *p* < 0.005] such that the (reverse) validity effect showed only in the Belief group (compare Figures 1, 2).

**Figure 3:**
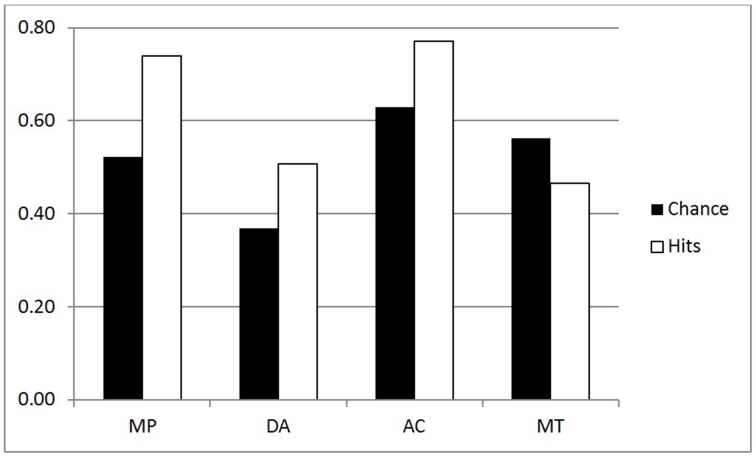
**Coherence analysis for the Inference group (all conditionals)**.

**Figure 4:**
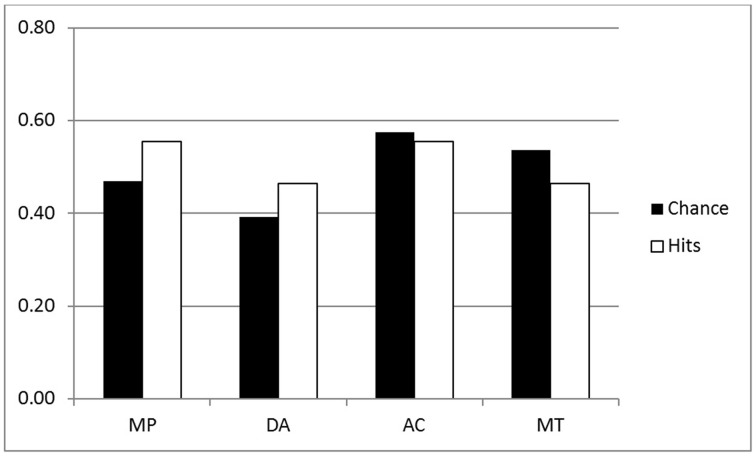
**Coherence analysis for the Belief group (all conditionals)**.

We performed an ANOVA for the coherence hit rates (all conditionals) with the same factors—see white bars of Figures 3, 4. There were three significant main effects: Group [*F*_(1, 44)_ = 17.02, MSE = 0.567, η^2^_*p*_ = 0.279, *p* < 0.001], as predicted with higher hit rates for the Inference group (0.62) than the Belief group (0.51); Validity [*F*_(1, 44)_ = 12.88, MSE = 0.016, η^2^_*p*_ = 0.226, *p* < 0.001]—again higher scores for invalid (0.58) than valid (0.56) inferences, and very large effect of Polarity [*F*_(1, 44)_ = 52.74, MSE = 0.363, η^2^_*p*_ = 0.545, *p* < 0.001] reflecting more hits for affirmative (0.66) than negative (0.48) inferences. A Validity by Group interaction [*F*_(1, 44)_ = 12.88, MSE = 0.016, η^2^_*p*_ = 0.226, *p* < 0.001] indicated that the (reverse) validity effect was detected only for the Inference group (the opposite trend to that shown in the p-validity analysis). Finally there was an interaction for Polarity by Group [*F*_(1, 44)_ = 13.07, MSE = 0.363, η^2^_*p*_ = 0.229, *p* < 0.001] reflecting a larger effect of Polarity in the Inference than the Belief group.

Before discussing these findings, it is important to consider the chance rates for assigning correct conclusion probabilities which we do next.

#### Chance rates

Uncertain deduction presents measurement problems unknown to the standard deduction paradigm. With the old method each conclusion is either valid or not and hence each response either correct or not. With the new method, however, a correct response or “hit” is a value lying within an interval: [minP, 1] for p-validity and [minP, maxP] for coherence. Moreover, these ranges depend upon not only the logical inference under consideration but the actual probabilities assigned to the premises by a particular participant on a particular problem.

The size of these ranges varies considerably and hence the participant has a high chance of guessing the correct answer when they are large. As pointed out in the introduction, where there is low belief in the premises, minP for p-validity may be set to 0, so that *any conclusion probability* will be deemed a hit. For these reasons, it seems essential to consider chance rates and to provide analyses which correct for them^2^. We decided to use the range of the target interval as a measure of chance level responding. For example, with p-validity, if minP was 0.4, we took the value 1-minP = 0.6 to be the chance rate. This is because any participant generating random probabilities with a uniform distribution between 0 and 1, would have a 0.6 chance of hitting the correct interval. For coherence, we took the value (maxP—minP) to be the chance rate for similar reasons. Hence, like hit rates, chance rates have to be computed for each individual participant, conditional and inference. The mean computed chance rates are shown as black bars in Figures 1–4.

The first question is whether the observed hit rates were above chance. To assess this, we first computed for each participant the mean difference between hits and chance scores for each conditional sentence, for each inference in both groups on both measures. We then compared these values to a mean of zero with a one sample t test (two tailed, df = 22) in each case. Considering first p-validity, as one might expect from Figure 1, scoring was highly significantly above chance for MP and AC in the p-validity analysis of the Inference group. Neither DA nor MT were significantly different from chance. For the Belief group (Figure 2) hits were again significantly above chance for MP and AC but significantly *below* chance for MT. In the coherence analysis for the Inference group (Figure 3) scores were (significantly) above chance for MP, DA, and AC but below chance for MT. In the Belief group (Figure 4) all differences were significant with scores above chance for MP and DA and *below* for AC and MT.

Overall, scores were above chance in the majority of cases, but with exceptions. In particular scores for MT tended to be below chance. The high chance rates clearly complicate the interpretation of the analyses of hits reported above. Hence, we decided to repeat these analyses using chance corrected scores, so that the value (hits-chance) was entered as the dependent variable. We refer to these as *performance scores*.

#### Chance corrected ANOVAs

##### Analysis of p-validity

An analysis of variance of was run on the performance scores (hits—chance) for both groups combined on the reduced set of 24 sentences. The factors were Group (Belief vs. Inference), Polarity (MP, AC vs. DA, MT), and Validity (MP, MT vs. AC, DA). All three main effects were statistically significant, the largest being polarity [*F*_(1, 44)_ = 132.25, MSE = 1.198, η^2^_*p*_ = 0.750, *p* < 0.001], indicating that performance was better on affirmative inferences (MP, AC; mean 0.138) than negative inferences (DA, MT; mean—0.023) as is evident from Figures 1, 2 when hits and chance are compared. There was a significant effect of Group [*F*_(1, 44)_ = 17.95, MSE = 0.200, η^2^_*p*_ = 0.290, *p* < 0.001], showing, as predicted, better performance in the Inference (0.090) than Belief group (0.025). Validity [*F*_(1, 44)_ = 10.58, MSE = 0.031, η^2^_*p*_ = 0.194, *p* < 0.002] was also significant, as performance was poorer on valid (0.044) than invalid (0.070) inferences due to reversal on MT. There were two significant interactions, one of which was relatively large: Polarity by Group [*F*_(1, 44)_ = 9.74, MSE = 0.088, η^2^_*p*_ = 0.181, *p* < 0.003]. It is evident from the Figures that the Polarity effect was substantially attenuated in the Belief group. There was also a small but significant three way interaction between Group, Polarity and Validity [*F*_(1, 44)_ = 4.24, MSE = 0.008, η^2^_*p*_ = 0.088, *p* < 0.05].

##### Analysis of coherence

Coherence tests apply regardless of the believability of the conditional statement, and so for this measure we report analyses of all 48 sentences. Chance and hit rates are shown on this measure in Figures 3, 4 for the Inference and Belief groups respectively. For the Inference group, performance appears to be well above chance for MP, DA, and AC but below chance for MT. Performance appears lower generally in the Belief group but the reverse trend for MT is still present.

The ANOVA for performance scores produced three large effects: Group [*F*_(1, 44)_ = 16.63. MSE = 0.523, η^2^_*p*_ = 0.437, *p* < 0.001] with higher scores for Inference (0.091) than Belief (0.016); Polarity [*F*_(1, 44)_ = 47.87, MSE = 0.332, η^2^_*p*_ = 0.521, *p* < 0.001 with higher scores for MP, AC (0.096)] than for DA, MT (0.011); and Validity by Polarity [*F*_(1, 44)_ = 167.95, MSE = 1.230, η^2^_*p*_ = 0.792, *p* < 0.001]. The main effect of Polarity and its interaction with Validity reflect the fact that performance reversed on MT for both groups (see Figures 3, 4). Also significant in this analysis were Validity [*F*_(1, 44)_ = 7.59, MSE = 0.033, η^2^_*p*_ = 0.147, *p* < 0.01], Polarity by Group [*F*_(1, 44)_ = 18.77, MSE = 0.130, η^2^_*p*_ = 0.299, *p* < 0.001] and Group by Validity by Polarity [*F*_(1, 44)_ = 8.82, MSE = 0.065. η^2^_*p*_ = 0.167, *p* < 0.01]. The validity effect is also due to poor performance on MT. The three way interaction reflects the fact that the Group by Validity interaction was more marked in the Inference group where performance on inferences other than MT was higher.

#### Statement probabilities

As indicated above, chance calculations depend upon the probabilities participants assign to the premises of each argument. Hit rates depend on the conclusion probability assigned. To aid in interpretation of the above findings, we examined the ratings of these statements directly. First, we looked at major premises—the conditional statements themselves. We checked for the Inference group whether conditionals were rated differently on the four occasions they appeared (with each inference). They did not, mean scores being almost identical. We compared the average of these with the single ratings of the same conditionals in the Belief group and they were again similar: Inference 45.2 (SD 19.1), Belief 47.3 (SD 23.4). A t test conducted across the 48 sentences showed no significant difference (*t* = 0.62).

Then we considered the ratings of the events p, not-p, q and not-q which comprise the minor premises and conclusions for the arguments. In the Belief group these are only rated once, but in the Inference group each is rated twice, once when acting as a premise (e.g., p for MP) and once as a conclusion (e.g., p for AC). Ratings as premises and conclusion were again extremely similar in all cases. There were however, substantial differences in the ratings given to affirmative events (p and q) with a mean of 0.52 and for negative events (not-p and not-q) with a mean of 0.39. This effect was very large as shown by an ANOVA [*F*_(1, 44)_ = 82.53, MSE = 0.324, η^2^_*p*_ = 0.652, *p* < 0.0001]. There was also marginally significant (*p* < 0.06) trend for antecedent events (0.48) to be rated higher than consequent events (0.45).

**Figure 5:**
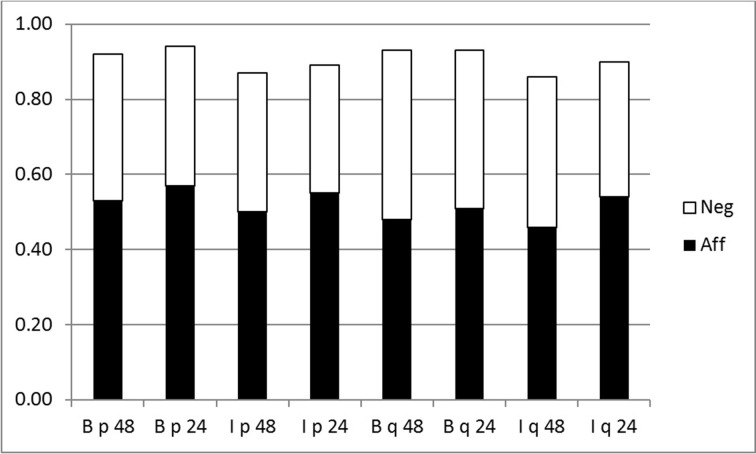
**Stacked bar chart showing probabilities assigned to events and their negations**. B, Belief group; I, Inference group; 48, full set of conditionals; 24, reduced set; p, antecedent event (black bar p, white bar not-p); q, consequent event (black bar q, white bar not-q).

It is important to note that ratings of affirmative and negative events were incoherent, i.e., inconsistent with probability theory. As Figure 5 illustrates, the sum of events and their negations was less than one in all cases, whether calculated for the full sets of 48 conditionals or the reduced set of 24. This incoherence has important implications for our findings. In the p-validity analyses (Figures 1, 2) chance rates were significantly higher for DA and MT which make use of negated events. This would follow from underestimation of negative events, because as we have shown earlier, lower belief in premises results in larger ranges for hits on this measure. It also affects chance rates for coherence measures (Figures 3, 4) but in the opposite direction. If assignments were coherent, then we would compute the same chance rates for MP and DA and the same for AC and MT. The former pair use *P(p)* and 1—*P(p)*, which should add to one, the latter *P(q)* and 1—*P(q)* which should also add to one.

To see why underestimating negative event probabilities reduces chance scores for the coherence measure, we take an example. Suppose for a particular conditional a participant sets *P(q|p)* = 0.7, *P(p)* = 0.6 and *P(not-p)* = 0.32. This shows the typical bias in our experiment, as *P(p) + P(not-p)* = 0.92 overall. When we compute the hit interval for MP, by the equations given in Table 1, we get [0.42, 0.82] with a chance calculation of 0.40. Had *P(not-p)* been assigned coherently, i.e., to 0.4, the interval for DA would compute to be [0.12, 0.58] again with a chance rate of 0.40. However, it is underestimated, resulting in a computed interval of [0.476, 0.796] and a chance rate of 0.32 which is lower than is should be.

## Discussion

The objective of the present study was to investigate the accuracy with which people can make probability judgments about the premises and conclusions of conditional inferences, and to test whether this accuracy, as measured by Bayesian standards, is increased by explicit conditional reasoning. We appealed to the standards of p-validity and coherence. We used two methods: a Belief group who rated beliefs in the statements presented separately, and an Inference group who saw them grouped as an explicit inference. We found that our participants did conform to p-validity at rates significantly higher than chance, but only for the affirmative inferences MP and AC. This performance was also significantly higher for the Inference group. Results were similar for the coherence measure. Again performance was well above chance for MP and AC, and significantly better for the Inference group. However, the results for the denial inference DA and MT were more complex, as participants were above chance for the former and below chance for the latter.

As must be evident to the reader, the study of uncertain deduction is a good deal more complex than use of the traditional deduction paradigm. In the traditional method, each inference is classified as valid or invalid and the participant either does or does not endorse the inference. To study uncertain deduction we must allow participants to assign probabilities to the premises and the conclusions of deductive arguments. The difficulty then comes in assessing whether they have done this correctly. First, there is not one but two different measures that can be taken: p-validity and coherence. Second, each of these allows participants to assign a conclusion probability within an interval. This interval must be computed for each participant on each problem separately depending on the premise probabilities assigned. Finally, these intervals can be large, creating the problem that participants may hit them by chance. We have shown in this paper how to compute these chance intervals and proposed method to correct hits rates for guessing.

Very little previous work has been conducted on uncertain deduction, despite apparent enthusiasm for a new paradigm psychology of reasoning based on degrees of belief rather than black and white truth judgments. The methodology introduced here differs in significant ways from the study of Pfeifer and Kleiter (2009, 2010) who studied only coherence (not p-validity), using premise probabilities assigned by the experimenter and allowing participants to assign a range of probabilities to the conclusion. Their results differ from ours in that they found coherence to be good only for MP, whereas we find this to be the case for MP, AC, and DA. This could reflect the difference in response method, but we think it more likely due to our use of realistic, causal-temporal conditional statements which introduce real world experience of causal relations. (We have no account of the reversal on MT, however.) In addition to assessing the coherence of conclusion probabilities taken as point ratings, we believe this to be the first psychological study to measure directly whether people conform to p-validity when both major and minor premises are taken to be uncertain. In both cases, this means that a range of values are acceptable as a “hit” on either measure. We consider our two measures in turn.

Probabilistic validity, or p-validity, is a relatively weak measure for us. For generality and to minimize our assumptions, we did not presuppose that *P(if p then q) = P(q|p)* in our assessment of p-validity, but simply assessed whether participants express no more uncertainty in the conclusion than in the premises of our conditional inferences. This notion of validity does not constrain conclusion probabilities for the invalid inferences, AC and DA, nor in effect, for valid inferences with low belief premises. Hit rates generally exceeded chance in our study only for the affirmative inferences MP and AC. Chance rates are disturbingly high (black bars, Figures 1, 2) even with the analysis restricted to the higher belief conditionals. Hence, we suggest that this measure will only be useful for problems where there is a very high degree of belief in the premises. Nevertheless, we have some findings of interest on this measure. First, as predicted, p-validity scores are higher for the Inference than the Belief group, with and without chance correction. The second finding of particular interest is that participants did not conform more to p-validity on the inferences that are actually valid, MP and MT. Indeed there was a small trend in the opposite direction. Much larger was an effect of polarity such that participants performed better on the affirmative inferences, MP and AC.

These findings can be accounted for as follows. First, the chance rates are very high on DA and MT due to underestimation of negative event probabilities, as explained earlier. This creates a ceiling effect for these two inferences, making it difficult for participants to perform above chance. This does not explain, however, why performance is equally high on MP and AC and facilitated for the Inference group in both cases. Research in the traditional paradigm often showed high endorsement of AC, though it is both classically invalid and p-invalid (Evans and Over, 2004). It may be that the participants interpreted the assertion of our causal-temporal conditionals (in Supplementary Material) *if p then q* as also pragmatically implying *if q then p*, making AC in effect MP in the other direction. That could explain their apparently equal effort to generate a p-valid conclusion in the Inference group for AC as for MP. In a study corresponding to our Inference group, Singmann et al. (2014) assessed p-validity only for MP and MT, and found that participants conformed to p-validity for MP and not MT. Still, as we have explained above, pragmatic factors can have a large effect people's reasoning. There could be pragmatic differences in the materials used by Singmann et al. and ourselves, and further research must investigate this possibility.

It could also be suggested that our use of causal-temporal conditionals, *if p then q*, implies not only that *P(q|p)* is high but that *P(q|not-p)* is low, in conformity with the *delta-p rule*, *P(q|p)*—*P(q|not-p)*, which measures how far that *p* raises the probability of *q*. It is true that, when *p* causes *q*, *p* would normally be thought to raise the probability of *q*, but previous work has not found that people interpret causal-temporal conditionals in terms of the delta-p rule (see Over et al., 2007, and especially Singmann et al., 2014, on this rule).

Use of the coherence measures allows us to ask whether people are coherent in the beliefs they express about conditional statements and their component events. This measure is stronger than that of p-validity and is applicable to both p-valid and p-invalid inferences. But the equations we use for coherence do assume that *P(if p then q) = P(q|p)*, which, as we explained above, is often called the Equation (Edgington, 1995). Examining the data, we have found again that coherence is better for the Inference than the Belief group, again with and without chance correction. As with p-validity, these analyses are affected by the underestimation of negative event probabilities, which in this case causes chance rates to drop somewhat for DA and MT. But it is striking that the facilitation of coherence in the Inference group is restricted to MP, DA, and AC, as can be seen by comparing Figures 3, 4 (see yet again Singmann et al., 2014, and recall our point about possible pragmatic differences between their materials and ours).

Interpretation of findings on the negative inferences, DA and MT, is clearly complicated by the underestimation of negative event probabilities we have observed. If we focus our attention on the affirmative inferences, MP and AC, however, it is clear that participants perform well above chance on both measures in the Inference group. In other words when given the opportunity to see the statements grouped as an inference, untrained participants do seem to grasp intuitively the logical restrictions that premise probabilities place upon conclusion probabilities. The actual hit rates are well over 80% for p-validity and around 75% for coherence. We find these figures quite encouraging, as supporting the conclusion that one way to improve Bayesian reasoning is by the use of explicit inferences. Explicit reasoning may not always make people rational by Bayesian standards, but it can help (see also Cruz et al., 2015).

Uncertain deduction is central to the new paradigm psychology of reasoning. If research is to progress, we must find methods for studying the relation between belief in premises and belief in conclusions. It is, as we have shown, a much trickier task than that presented by the standard deduction paradigm. There are a number of pointers to future research studies arising from our findings. For example, studies of p-validity should be restricted to problems with high belief (but still uncertain) premises, in order to provide sufficient sensitivity. We have also highlighted a problem with explicitly negated premises. Events expressed as negations tend to be underestimated in their probabilities, providing an immediate source of incoherence. This could be related to the findings in “support theory” of *subaddivity*: that the weight given to an implicit disjunction is less than the sum of its disjuncts when these are made explicit (Tversky and Koehler, 1994). A negated event is itself an implicit disjunction; that is, not-A consists of B v C v …, which are the explicit alternatives. For example, the probability assigned to “school class sizes are not reduced” might be less than the sum that would be assigned to “school class sizes are increased” and “school class sizes remain the same.” In any event, this problem must be addressed in future studies of the coherence of negated inferences^3^.

We believe that there is much to be gained from the further study of the coherence of conditional beliefs, as in our Belief group. We have noted above the rich literature that resulted from the discovery of the conjunction fallacy. The representativeness heuristic that Tversky and Kahneman (1983) proposed as an explanation of this incoherence in conjunctive beliefs might also cause some incoherence in conditional beliefs, but other, as yet unknown heuristics could play a role as well. We hope to have demonstrated here, however, that the study of deductive reasoning using Bayesian methods should move beyond the almost exclusive focus on the inference from *p and q* to *q* and the associated conjunction fallacy. There is much more to discover about Bayesian reasoning by studying other deductive inferences with uncertain premises.

In an ideal Bayesian world, probabilities assigned to logically related statements would be perfectly coherent with probability theory, but in reality this is unlikely to hold, especially when the statements are not explicitly related as inferences. Such probabilities are unlikely to be assigned on an absolute basis due to the power of pragmatics in human communication and understanding. We interpret statements in their context, amplifying their meanings and making probability judgments with implicit heuristics. It is unsurprising that people's beliefs are not fully coherent. It is impossible for them to ensure absolute coherence, even in relatively simple beliefs, due computationally intractability. However, it is of great interest to discover the causes of incoherence in conditional beliefs, such as the difficulty with negative events reported here.

Grouping uncertain statements together as an inference is a natural way to extend the traditional deduction paradigm to the study of uncertain deduction. The fact that participants in our Inference group consistently performed better than participants in the Belief group might suggest that the former were intervening with explicit reasoning in order the make their judgments more consistent. Further research will be needed, however, to determine whether this is in fact that case. An alternative pragmatic account is that concurrent presentation of premises and conclusions contextualizes the statements together so that judgments become more consistent without any conscious effort of reasoning. If explicit reasoning is involved, this could be indicated by examining performance under working memory load or by correlating performance with individual measures of cognitive ability. These are among the methods employed by dual process researchers to identify effortful reasoning (Evans and Stanovich, 2013).

In conclusion, we hope to have developed a methodology that can be adapted for a variety of future uses in the new psychology of deduction. We have shown that it is feasible to study the relation between the degree of belief that people hold in premises and conclusion of a logical argument. We have also shown that such judgments are not random and conform to the coherence of probability theory at rates well above that which could be expected by chance. People have some problems with the coherence of their judgments about negative events, but are otherwise fairly good, by Bayesian standards, at conditional reasoning. Their performance is at an even higher level when statements are grouped together into explicit conditional inferences.

### Conflict of interest statement

The authors declare that the research was conducted in the absence of any commercial or financial relationships that could be construed as a potential conflict of interest.

## Footnotes

^1^A related, but extensional, definition of deduction is that the conclusion cannot convey more semantic information than the premises (Johnson-Laird, 1983).

^2^We thank Phil Johnson-Laird for alerting us to this problem.

^3^The coherence intervals of Table 3 are derived using the total probability theorem of probability theory. See Hadjichristidis et al. (2014) on this theorem and their findings of superaddivity.
