# Supplementary material for: Uncertain deduction and conditional reasoning
Source: Front Psychol. 2015 Apr 8;6:398. doi: 10.3389/fpsyg.2015.00398 (PMC4389288; doi:10.3389/fpsyg.2015.00398)
Supplement: Supplementary file 1 [file DataSheet1.DOCX]

**APPENDIX A**

The 48 conditionals sentences used in the study, together with belief ratings from Belief group

(Sentences 1 – 24 used as the reduced set with higher mean belief ratings)

|  |  |  |
| --- | --- | --- |
| 1 | If oil prices continue to rise then gas prices in Canada will rise | 73.7 |
| 2 | If Saskatoon continues to grow then traffic jams will get worse | 77.7 |
| 3 | If more people use sun screen then cases of skin cancer will be reduced | 74.2 |
| 4 | If people continue hunting without licenses then more animals will become extinct | 55.7 |
| 5 | If the weather gets warmer more people will go to the beach | 60.8 |
| 6 | If Sony release a new PlayStation console then their company profits will rise | 59.8 |
| 7 | If drunk driving is reduced then there will be fewer accidents | 69.2 |
| 8 | If newspaper subscriptions drop then there will be fewer paper routes | 71.3 |
| 9 | If immigration laws are made stricter then the number of immigrants in Canada will decrease | 62.3 |
| 10 | If more parents read to their children then literacy rates will improve | 65.4 |
| 11 | If jungle deforestation continues then gorillas will become extinct | 61.7 |
| 12 | If fertility treatment improves then the world population will rise | 57.3 |
| 13 | If more people exercise then rates of heart disease will decrease | 67.2 |
| 14 | If Adidas get more athletes to wear their running shoes then their sales will increase | 72.5 |
| 15 | If city transit is improved then fewer people will drive their cars to work | 56.9 |
| 16 | If healthy foods become cheaper then more people will eat healthily | 69.3 |
| 17 | If tuition is increased then applications for the University of Saskatchewan will drop | 49.4 |
| 18 | If nurses’ salaries are improved the recruitment of nurses will increase | 62.0 |
| 19 | If student loans’ interest is decreased then university entries will increase | 54.9 |
| 20 | If Canada decreases greenhouse gas emissions then global warming will be reduced | 52.5 |
| 21 | If elementary school class sizes are reduced then national literacy will improve | 45.7 |
| 22 | If American troops remain in Iraq then acts of terrorism in the US will increase | 58.6 |
| 23 | If genetic research continues then a cure for cancer will be found | 50.3 |
| 24 | If conscription is reintroduced then anti-social behaviour will decrease | 26.0 |
|  |  |  |
| 25 | If traffic is reduced then more children will walk to school | 43.2 |
| 26 | If violence is reduced on television then the amount of violent crime will be reduced | 39.8 |
| 27 | If divorce is made more difficult then the number of marriages will decrease | 48.1 |
| 28 | If unemployment rates drop then Harper will win the next election | 41.2 |
| 29 | If foreign potash investment is encouraged then the SK potash industry will flourish | 60.6 |
| 30 | If the Conservative Party changes their leader then they will win the next election | 33.9 |
| 31 | If space exploration continues then extra-terrestrial beings will be discovered | 37.3 |
| 32 | If minor league hockey facilities are improved then Canada will win the Stanley Cup | 40.7 |
| 33 | If summer schools are made mandatory then applications to University will decrease | 31.3 |
| 34 | If parenting is taught in schools then juvenile crime rates will increase | 23.2 |
| 35 | If more new houses are built then the amount of homeless people will increase | 28.0 |
| 36 | If third world debt is canceled then world poverty will worsen | 20.9 |
| 37 | If fast food is taxed then childhood obesity will increase | 21.0 |
| 38 | If the number of safe injection sites in SK are increased then HIV will spread AB | 29.8 |
| 39 | If sales of online books increase then there will be more libraries | 17.8 |
| 40 | If the lottery prize-money increases then fewer people will buy tickets | 21.0 |
| 41 | If you buy fewer luxuries then you will save less money | 23.6 |
| 42 | If more anti-venom is produced then more people will die from snake bites | 22.8 |
| 43 | If global temperatures rise then less arctic ice will melt | 18.8 |
| 44 | If electric cars become widely-used then air pollution will worsen | 24.2 |
| 45 | If people recycle more then there will be more demand for landfills | 27.1 |
| 46 | If the price for cable TV increases then more people will sign up to receive cable | 20.1 |
| 47 | If people quit smoking then they will die earlier | 16.1 |
| 48 | If elementary school class sizes are reduced then national literacy will worsen | 25.4 |
